# Prolapsus Vaginæ in the Bitch; Luxatio Femoris in a Lion; Nymphomania in a Castrated Female Cat

**Published:** 1900-07

**Authors:** Cecil French

**Affiliations:** Washington, D. C.


### DEPARTMENT OF CANINE AND FELINE MEDICINE AND SURGERY.

### PROLAPSUS VAGINÆ IN THE BITCH; LUXATIO FEMORIS IN A LION; NYMPHOMANIA IN A CASTRATED FEMALE CAT.

By Cecil French, D.V.S.,
WASHINGTON, D. C.

Prolapsus Vaginæ in the Bitch commonly occurs as a protrusion of a congested portion of the vaginal mucosa associated with the advent of sexual excitement. It tends to occur periodically, and is then described as being habitual. It usually subsides with the departure of the heat, so that operative measures must not be considered imperative. It being an objectionable blemish, though only temporary, owners usually prefer to have the enlargement removed by the surgeon.

Removal may be effected by employment of crucial ligature with excision by the knife, by clamp and cautery, or by écraseur or emasculator.

The crucial ligature method is attended with an element of risk on account of hemorrhage, owing to the tendency ligatures have to slip from this character of tissue. The clamp and cautery method is effective in controlling hemorrhage but leaves an unnecessary area of scar-tissue. The écraseur lacerates the tissues considerably and is very apt to be followed by hemorrhage.

Removal by emasculator (small size) *with subsequent suture of the divided mucosa* must be given the preference for the reason that *simple* excision of a large prolapsus leaves a considerable area of tissue denuded of mucosa from which passive hemorrhage is prone to occur, owing to the physiological turgescence of the organs during the heat period.

The method I have found most satisfactory is as follows: A strong suture is passed through the substance of the tumor, by which the latter is withdrawn well out of the vagina. The urethral orifice is now sought and particular care exercised not to include the same in the adjustment of the instrument. After the instrument has been made to sever the prolapsed portion and before it is removed, a continuous silk suture is run through the approximated edges of the divided mucosa immediately beneath the clamped emasculator. The stitch is commenced furthest from the vulva and its nearest end is left long enough to hang out of the vagina, by which it can be removed in the course of five or six days.

Luxatio Femoris in a Lion. This animal is an inmate of the National Zoological Park. It had been lame in the right hind-leg for some two weeks. Close inspection through the bars of the cage showed the existence of a swelling over the hip-joint and an apparent shortening of the affected leg.

The keepers secured the animal by their usual methods and bound its fore-parts firmly to the bars. The door was then opened and the hind-quarters secured with ropes and drawn through.

Manipulation revealed a dislocation of the head of the femur. The latter was resting on the upper margin of the acetabulum.

Reduction of the luxation was effected in the following manner: Forcible traction was produced by two men pulling on the rope attached to the lower part of the leg, the latter being extended and at the same time abducted. Further assistance was rendered by one of the attendants exerting pressure over the trochanter with his heel. The animal apparently suffered much pain during the operation. Recovery was complete within three weeks. The injury was supposed to have been brought about by the animal striking its hip against the ironwork and slipping in its passage through a door from one cage to another.

Nymphomania in a Castrated Female Cat. In March, 1899, I removed from the abdominal cavity of a pregnant female cat both ovaries, oviducts, and the entire uterus, the latter containing six fetuses. A month later the animal exhibited a desire for sexual intercourse, and has been in the same condition ever since—a period of fourteen months. The owner refuses to permit me to conduct another laparotomy for explorative purposes, so that I am unable to satisfy myself as to whether I succeeded in removing both ovaries in their entirety or not. The fact that the "heat" in this case has been continuous would seem to indicate the presence of some morbid condition of the nervous system and not the functioning of a portion of an ovary remaining, in which case the "heat," as a rule, appears periodically at the usual season. The owner has never been able to catch this animal in coition, but her constant crying and the attentions of numerous members of the opposite sex leave no doubt as to her condition.

